# Biological nitrogen fixation in the long-term nitrogen-fertilized and unfertilized paddy fields, with special reference to diazotrophic iron-reducing bacteria

**DOI:** 10.1007/s00203-023-03631-8

**Published:** 2023-07-20

**Authors:** Yoko Masuda, Sakura Satoh, Ryota Miyamoto, Ryo Takano, Katsuhiro Ishii, Hirotomo Ohba, Yutaka Shiratori, Keishi Senoo

**Affiliations:** 1 Department of Applied Biological Chemistry, Graduate School of Agricultural and Life Sciences, The University of Tokyo, 1-1-1 Yayoi, Bunkyo-City, Tokyo, 113-8657 Japan; 2 Niigata Agricultural Research Institute, 857 Nagakuramachi, Nagaoka, Niigata 940-0826 Japan; 3 Collaborative Research Institute for Innovative Microbiology, The University of Tokyo, 1-1-1 Yayoi, Bunkyo-City, Tokyo, 113-8657 Japan

**Keywords:** Paddy soil, Iron-reducing bacteria, Nitrogen fixation, Nitrogen fertilization, Long-term field experiment

## Abstract

Biological nitrogen fixation (BNF) is important to sustain nitrogen fertility of paddy soil and rice yield, while could be affected by nitrogen fertilization. Iron-reducing bacteria, *Anaeromyxobacter* and *Geobacter*, are newly found diazotrophic bacteria predominant in paddy soil. Experimental field of this study is a long-term (35 years) nitrogen fertilized (6.0 g N/m^2^/year) and unfertilized paddy field, where ca. 70% of rice yield was obtained yearly in nitrogen unfertilized plot (443 ± 37 g/m^2^) compared to fertilized plot (642 ± 64 g/m^2^). Effects of long-term nitrogen fertilization/unfertilization on soil properties related to BNF were investigated with special reference to diazotrophic iron-reducing bacteria. Soil chemical/biochemical properties, soil nitrogen-fixing activity, and community composition of diazotrophic bacteria were similar between nitrogen fertilized and unfertilized plot soils. In both plot soils, *Anaeromyxobacter* and *Geobacter* were the most predominant diazotrophs. Their *nifD* transcripts were detected at similar level, while those of other general diazotrophs were under detection limit. It was concluded that long-term use/unuse of nitrogen fertilizer in this field did not affect the predominance and nitrogen-fixing activity of diazotrophic iron-reducing bacteria, composition of other general diazotrophs, and the resulting soil nitrogen-fixing activity. BNF, primarily driven by diazotrophic iron-reducing bacteria, might significantly contribute to sustain soil nitrogen fertility and rice yield in both plot soils. Appropriate soil management to maintain BNF, including diazotrophic iron-reducing bacteria, will be important for sustainable soil nitrogen fertility and rice production.

**Supplementary Information:**

The online version contains supplementary material available at 10.1007/s00203-023-03631-8.

## Introduction

Nitrogen is an essential nutrient for plant growth and is generally applied to soil to obtain high crop yields. In upland crop fields, crop yields are significantly lower when cultivated under nitrogen-free fertilization (Zhou et al. 2011). In contrast, rice yields in paddy fields are maintained at relatively high levels even under nitrogen-free fertilization (Okumura 2015), indicating that soil nitrogen fertility plays a significant role in supporting paddy rice growth and that paddy soils have unique mechanisms to sustain nitrogen fertility.

Biological nitrogen fixation (BNF) is important to sustain soil nitrogen fertility, especially in paddy soil. Several studies demonstrated that nitrogen-fixing activity is remarkably higher in paddy soils than in other crop-field soils (Yoshida and Ancajas 1973; Ladha et al. 2016), accounting for about 30 kg N/ha/year (Herridge et al. 2008). Cyanobacteria, Alphaproteobacteria, Betaproteobacteria, and Gammaproteobacteria were considered to be key nitrogen fixing microorganisms in paddy soil (Mårtensson et al. 2009; Shapleigh 2006). However, our metatranscriptomic analysis suggested that iron-reducing bacteria (*Anaeromyxobacter* and *Geobacter*) were predominant drivers of nitrogen fixation in paddy field soil (Masuda et al. 2017). Their universal distribution and predominance in other paddy soils was also confirmed (Masuda et al. 2017). Our subsequent studies verified the nitrogen-fixing ability of iron-reducing bacteria isolated from paddy soils (Masuda et al. 2020; Xu et al. 2021). In addition, the enhancement of the nitrogen-fixing activity of laboratory and field paddy soils by applying iron oxide, an electron acceptor used by iron-reducing bacteria was demonstrated (Masuda et al. 2021). These results suggest the contribution of diazotrophic activity of iron-reducing bacteria in sustaining nitrogen fertility in paddy soil. However, these field studies were performed in a continuous experimental paddy field with conventional nitrogen fertilization (5 g N/m^2^/year) for 10 years. Several studies reported that nitrogen fertilization affected nitrogen fixation properties such as soil nitrogen-fixing activity and the nitrogen-fixing microbial community in paddy soil (Watanabe et al. 1981; Ishii et al. 2011; Tang et al. 2017). It is unclear whether the nitrogen-fixing microbial community dominated by iron-reducing bacteria in the study field soil originally existed or had been changed to some extent as affected by 10 year nitrogen fertilization.

Within the agricultural research institute, where these field investigations were conducted, long-term experimental field plots were established in 1984. Rice was cultivated yearly with or without nitrogen fertilization (6 g N/m^2^/year). In the no-nitrogen fertilization (NF) plot, approximately 70% of rice yield was obtained yearly compared to the standard nitrogen fertilization (SF) plot (mean annual yield of 1984–2018; NF = 443 ± 37 g/m^2^ and SF = 642 ± 64 g/m^2^). Nitrogen fixation would be important in sustaining nitrogen fertility to support the rice yield for 35 years, particularly in the NF plot soil. The NF and SF plot soils in this long-term experimental field are good research targets to examine whether iron-reducing diazotrophs are predominant drivers of nitrogen fixation in the soils, and whether long-term nitrogen fertilization caused any difference in nitrogen fixation properties between NF and SF plot soils. Consequently, a comparative analysis of both NF and SF plot soils was performed to determine the soil nitrogen-fixing activity, nitrogen fixation gene transcripts, and nitrogen-fixing microbial community composition, especially focusing on diazotrophic iron-reducing bacteria. The comparative analysis of chemical/biochemical properties and functional gene composition of both plot soils were also carried out.

## Materials and methods

### Experimental field and soil sampling

**Fig. 1:**
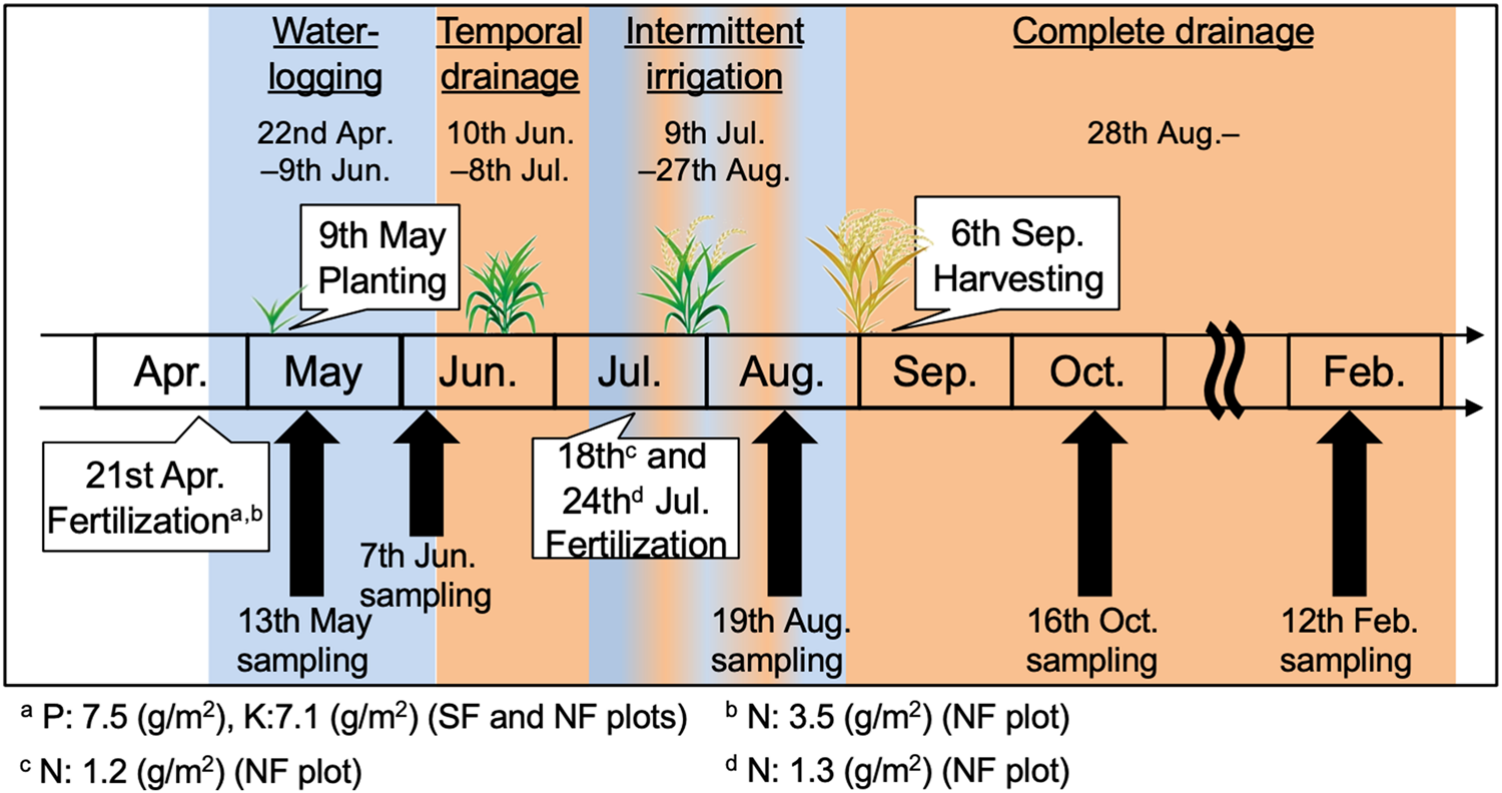
Annual field management in this study

The experimental paddy field for long-term fertilization was established in 1984 at the Niigata Agricultural Research Institute, Nagaoka, Niigata, Japan (37° 44ʹ N, 138° 87ʹ E), where rice (*Oryza sativa* L. Koshihikari) was cultivated. Every year in April, 3.5 g N/m^2^, 7.5 g P/m^2^, and 7.1 g K/m^2^ were applied as basal fertilization (ammonium chloride, ammonium phosphate, ammonium sulfate, multiphosphate, and potassium chloride) to the SF plot; in July, 1.2 and 1.3 g N/m^2^ were applied as additional fertilization (ammonium sulfate). To the NF plot, 7.5 g P/m^2^ and 7.1 g K/m^2^ were applied as basal fertilization (multiphosphate and potassium chloride) every year in April. The soil and crop management schedule in the field in 2019 is shown in Fig. 1. These activities were performed every year on a similar schedule. The physicochemical characteristics of both plot soils are listed in Table S1. Soil pH and soil redox potential (Eh) at a depth of 5 cm in the plow layer were measured using a pH/Eh meter with a sensor/platinum electrode (PRN-41; Fujiwara Co., Tokyo, Japan).

Soil samples were collected during the 2019 rice cultivation season at five different times: 3 and 6 weeks after the onset of waterlogging (13 May and 7 June 2019), at the end of intermittent irrigation (19 August 2019), and 1 and 5 months after harvesting during the complete drainage period (16 October 2019 and 12 February 2020; Fig. 1). Soil samples from a depth of ~ 5 cm were collected using headless plastic syringes (30 mL; with the head portion cut off). Syringes containing the soil samples were capped with butyl rubber stoppers. Three replicates of soil samples were collected from each plot. The soil samples for Fe^2+^, NH_4_^+^ and NO_3_^−^ concentration, biomass nitrogen (*B*_N_), and nitrogen-fixing activity measurements were transported to the laboratory at 4 °C and immediately subjected to tests. Soil samples for soil RNA/DNA extraction were frozen in liquid nitrogen immediately after collection, transported to the laboratory with dry ice, and stored at − 80 °C for subsequent use.

### Soil chemical/biochemical analysis

The Fe^2+^, NH_4_^+^ and NO_3_^−^ content of each soil sample were determined as described previously (Masuda et al. 2018; Ishii et al. 2011).

Biomass nitrogen (*B*_N_) measurement was performed according to the fumigation–extraction method. Each 5 g soil sample was subjected to (i) immediate extraction with 25 mL of 0.5 M K_2_SO_4_ (*B*_1_) and (ii) 24 h fumigation at CHCl_3_, followed by 0.5 M K_2_SO_4_ extraction (*B*_2_). The K_2_SO_4_ extract was filtered using paper filter (Advantec No. 6, Japan). A mixture of 1 mL filtered extract, 4 mL of 0.5 M K_2_SO_4_, and 5 mL of the oxidizing solution (Davi et al. 1993) was prepared and autoclaved at 120 °C for 30 min. Nitrate concentration was determined spectrophotometrically at a wavelength of 220 nm (Norman and Stucki 1981). The amount of *B*_N_ was calculated using the equation *B*_N_ = *B*_2_ − *B*_1_.

The nitrogen-fixing activity of each soil sample was measured using the acetylene reduction assay (Postgate 1972). Each 5 g soil sample was placed in 50 mL vials and sealed with butyl rubber and an aluminium crimp. The gaseous phase was exchanged with Ar/C_2_H_2_ gas (90:10, v/v). After incubation at 30 °C for 24 h, gas sample was collected from the gaseous phase of the vial, and the C_2_H_4_ concentration was measured using gas chromatography, as described previously (Masuda et al. 2020).

### DNA/RNA extraction and quantitative polymerase chain reaction (qPCR) analysis

Each 0.5 g (wet weight) of soil sample was subjected to DNA/RNA extraction, following the protocol by Angel et al. (2012). The extracted crude DNA/RNA solution was purified using a one-step PCR inhibitor removal kit (Zymo Research, Irvine, CA, USA). DNA/RNA quantity was determined using a Qubit 2.0 fluorometer (Invitrogen, Carlsbad, CA, USA) with a Qubit ds DNA HS assay kit and a Qubit RNA HS assay kit (Invitrogen), respectively. To obtain DNA solution, the cleaned DNA/RNA solution was treated with RNase A (TaKaRa Bio, Shiga, Japan). Removal of the remaining DNA from DNA/RNA solutions and cDNA synthesis was performed using the ReverTra Ace^R^ qPCR RT Master Mix with gDNA Remover (Toyobo, Osaka, Japan) according to the manufacturer’s protocol. The *nifD* gene, a more reliable indicator of diazotrophs than *nifH* gene, was used in this study to avoid detecting pseudo-*nifH* present in soil (Mise et al. 2021). Quantitative PCR (qPCR) was performed targeting *nifD* of general diazotrophs, *nifD* of *Anaeromyxobacter*/*Geobacter* in soil cDNA samples using each specific primer set (Masuda et al. 2021). The PCR conditions and standard curve for each target gene were prepared as described previously (Masuda et al. 2020, 2021).

### Shotgun DNA sequencing and informatics analysis

Shotgun sequencing of DNA samples prepared from triplicate soil samples collected on 7 June was performed using the DNBSEQ-T7 platform (MGI, Shenzhen, China) according to the manufacturer’s instructions for 2 × 200 paired-end at Seibutsugiken (Kanagawa, Japan). Paired-end sequences were joined together and converted from FASTQ to the FASTA format, as described previously (Masuda et al. 2018). The summary of metagenomic sequences, including deposit IDs, is shown in Table S2. Functional gene sequences were determined using a BLAT search against the Refseq database (*E* value < 10^−5^, alignment length of > 50 bp) on the MG-RAST server version 4.0.3 (Meyer et al. 2008). The retrieved sequences were compared to the nr database using BLASTX for a more accurate assignment (*E* value < 10^−5^, alignment length > 30 amino acids).

### Statistical analysis

Mann–Whitney *U* tests were performed using R software ver. 3.0.1 (R Core Team 2013) for the statistical analysis of soil chemical/biochemical properties, nitrogen fixing activities, and functional gene abundance.

## Results and discussion

Physicochemical characteristics of soils including total nitrogen collected before the rice cultivation season were similar between NF and SF plot (Table S1). In the NF and SF plots, a decrease in soil Eh and an increase in soil pH were observed during the waterlogged period, and a reverse trend was observed during the drained period (Fig. S1A). Soil Fe^2+^ concentration increased during the waterlogged period, indicating reduced soil condition in both plots (Fig. S1B). The transitions of these soil chemical properties were mostly similar between NF and SF plots and consistent with previous studies (Itoh et al. 2013; Masuda et al. 2017). B_N_ in the SF soil was significantly higher than in the NF soil in May and August but was similar in June and October (Fig. S1C). This could be attributed to applying nitrogen fertilizer to the SF plots in April and July (Fig. 1). Soil NH_4_^+^ would be generated from mineralization of soil organic matter including B_N_ (NF and SF plot soils) and applied fertilizer (SF plot soil), then absorbed by rice plant. Concentration of the resulting NH_4_^+^ in soil was similar between NF and SF plot soils throughout the sampling period (Fig. S1D). Nitrate was under the detection limit (< 0.02 μmol/g soil) in both plot soils throughout the sampling period (data not shown). Overall, no significant differences in chemical/biochemical properties, except for biomass nitrogen, between NF and SF soils were observed, indicating that 35 year use/unuse of nitrogen fertilizer at 6.0 g N/m^2^/year did not largely affect these properties.

**Fig. 2:**
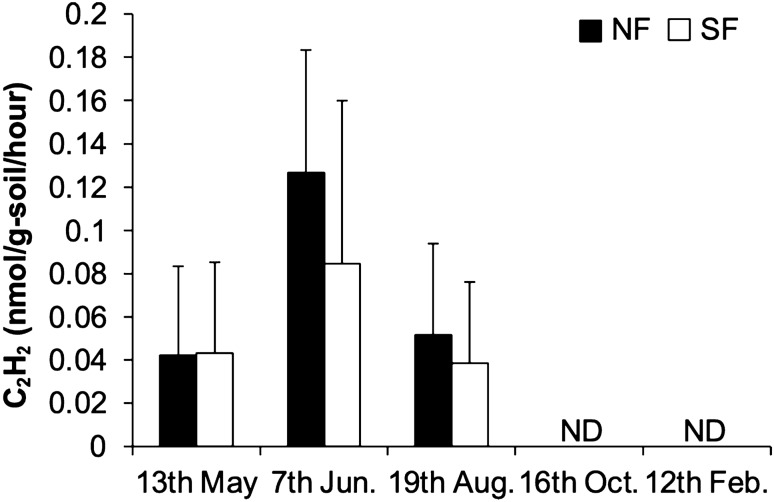
Transitions of the nitrogen fixation activity in Nagaoka paddy fields. *NF* no fertilization, *SF* standard fertilization

Soil nitrogen-fixing activity, measured using an acetylene reduction assay, was higher during the waterlogged period (May, June, and August) than during the drained period (October and February) in both plots and was not significantly different between NF and SF plots (Fig. 2). Soil Eh and Fe^2+^ concentration indicated the development of a reduced soil environment during the waterlogged period (Fig. S1A, B). These results suggest that anaerobic and/or microaerophilic microorganisms in a reduced soil environment mainly drive nitrogen fixation in both plot soils. Soil RNA analysis (RT-qPCR) was used to identify active nitrogen-fixing microorganisms in the soils. Transcripts of *nifD* derived from *Anaeromyxobacter* and *Geobacter*, anaerobic iron-reducing bacteria, were only detected in both plot soils at a similar level in June when soil nitrogen-fixing activity was at its maximum (Table 1). In contrast, *nifD* transcripts derived from other general diazotrophs were below the detection limit for both plot soils (Table 1). Iron-reducing bacteria might be significant drivers of nitrogen fixation among various diazotrophs in both NF and SF plots at least during the waterlogged period when iron reduction was in progress.

**Table 1:** Copy numbers of *nifD* gene transcripts in both plot soils

	NF			SF		
	13th May	7th Jun	19th Aug	13th May	7th Jun	19th Aug
*Anaeromyxobacter* and *Geobacter*	ND	(3.9 ± 3.8) × 10^6^	ND	ND	(5.8 ± 0.8) × 10^6^	ND
General diazotrophs	ND	ND	ND	ND	ND	ND

*NF* no fertilization, *SF* standard fertilization, *ND* under the detection limit

**Fig. 3:**
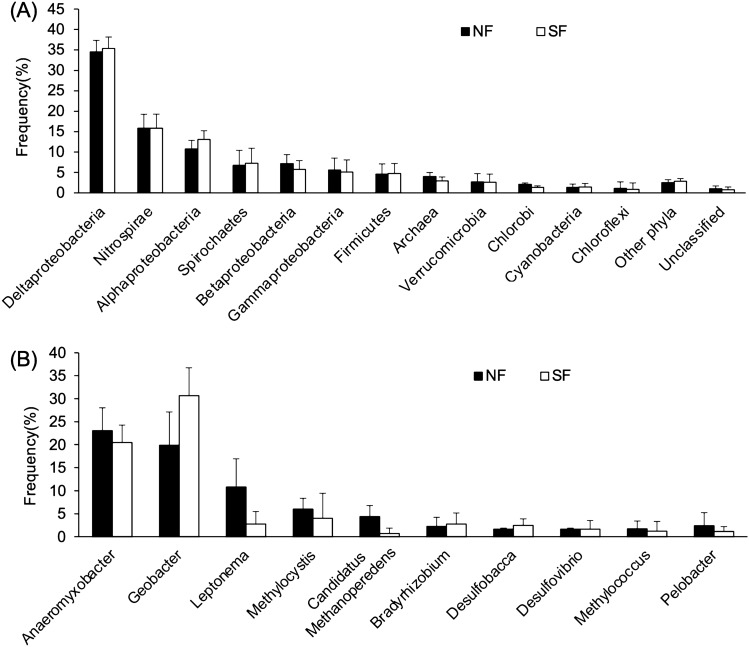
Composition of *nif* gene derived bacteria at phylum level (**A**) and predominant genera (**B**) based on metagenomic analysis. *NF* no fertilization, *SF* standard fertilization

Analysis of the *nif* gene in metagenome was performed to examine the compositions of nitrogen-fixing bacteria in soils from both plots collected on 7 June when soil nitrogen-fixing activity was at its maximum. As a result, the composition at the phylum/proteobacteria class and predominant genera level was similar between SF and NF plots (Fig. 3). In both plots, the *nif* gene derived from class Deltaproteobacteria was the most dominant at the phylum/proteobacterial class level (Fig. 3A), and *Anaeromyxobacter* and *Geobacter* were the most dominant genera (Fig. 3B). Results of the metagenomic *nif* gene analysis support the supposition that iron-reducing bacteria might be important drivers of nitrogen fixation in both plots and contribute to sustaining soil nitrogen fertility. In the functional gene analysis in the metagenome, the composition of functional gene categories was similar between plots (Fig. S2), suggesting that the long-term use/unuse of nitrogen fertilizer did not largely affect basic microbial functions.

Biological nitrogen fixation would be important in sustaining nitrogen fertility to support the rice yield for 35 years, particularly in the NF plot soil. This study indicated that nitrogen fertilization in SF plot soil at 6.0 g N/m^2^/year for 35 years did not cause a difference in the properties related to nitrogen fixation (soil nitrogen-fixing activity, active nitrogen-fixing bacteria, diazotrophic bacterial community composition, and predominant diazotrophs) compared to nitrogen unfertilized NF plot soil. It is thus suggested that biological nitrogen fixation in SF plot soil as well would contribute, to some extent, to sustain soil nitrogen fertility and rice yield. In both plot soils, diazotrophic iron-reducing bacteria (*Anaeromyxobacter* and *Geobacter*) might be significant drivers of nitrogen fixation and contribute to sustaining soil nitrogen fertility. In a long-term paddy field experiment in the Philippines with the same nitrogen fertilization level as in this study, no significant difference in nitrogen-fixing activity between long-term unfertilized and nitrogen-fertilized soils was found (Watanabe et al. 1981). In contrast, in a field experiment in China, a significantly lower nitrogen-fixing activity was observed in long-term (20 years) nitrogen-fertilized paddy soils than in unfertilized soils, where a thrice higher amount of fertilizer (18.2 g N/m^2^/year) than that used in this study was applied (Tang et al. 2017). The long-term nitrogen fertilization employed in this experimental field (6.0 g N/m^2^/year for 35 years) did not cause a difference in transition of soil NH_4_^+^ concentration between NF and SF plot soils (Fig. S1D), thus might not cause differences in the properties related to nitrogen fixation.

## Conclusions

Diazotrophic iron-reducing bacteria (*Anaeromyxobacter* and *Geobacter*), previously overlooked soil nitrogen-fixing bacteria, might be significant drivers of nitrogen fixation in both NF and SF plot soils of the long-term nitrogen fertilized/unfertilized paddy field and might contribute to sustain soil nitrogen fertility and rice growth. Thirty-five year use/unuse of nitrogen fertilizer at 6.0 g N/m^2^/year did not affect the predominance and nitrogen-fixing activity of diazotrophic iron-reducing bacteria, composition of other general diazotrophs, and the resulting soil nitrogen-fixing activity. As to nitrogen nutrient, rice yield in NF plot (443 ± 37 g/m^2^) would be supported largely by soil nitrogen fertility derived from biological nitrogen fixation, while increased rice yield in SF plot (642 ± 64 g/m^2^) would be supported by soil nitrogen fertility and additional nitrogen fertilizer. As such, biological nitrogen fixation is important to achieve sustainable rice production with minimal nitrogen fertilization. Appropriate soil management to maintain biological nitrogen fixation, including diazotrophic iron-reducing bacteria, will be important for sustainable soil fertility and rice production.

## Supplementary Information

Below is the link to the electronic supplementary material.

Supplementary file1 (PDF 66 KB)

Supplementary file2 (DOCX 19 KB)

## Data Availability

Sequence data derived from the metagenomic analyses in this study were deposited in the MG-RAST server (https://www.mg-rast.org). Deposit IDs are listed in Table S2.
