# Supplementary material for: Biological nitrogen fixation in the long-term nitrogen-fertilized and unfertilized paddy fields, with special reference to diazotrophic iron-reducing bacteria
Source: Arch Microbiol. 2023 Jul 20;205(8):291. doi: 10.1007/s00203-023-03631-8 (PMC10359436; doi:10.1007/s00203-023-03631-8)
Supplement: Supplementary file 1 — Supplementary file1 (PDF 66 KB) [file 203_2023_3631_MOESM1_ESM.pdf]

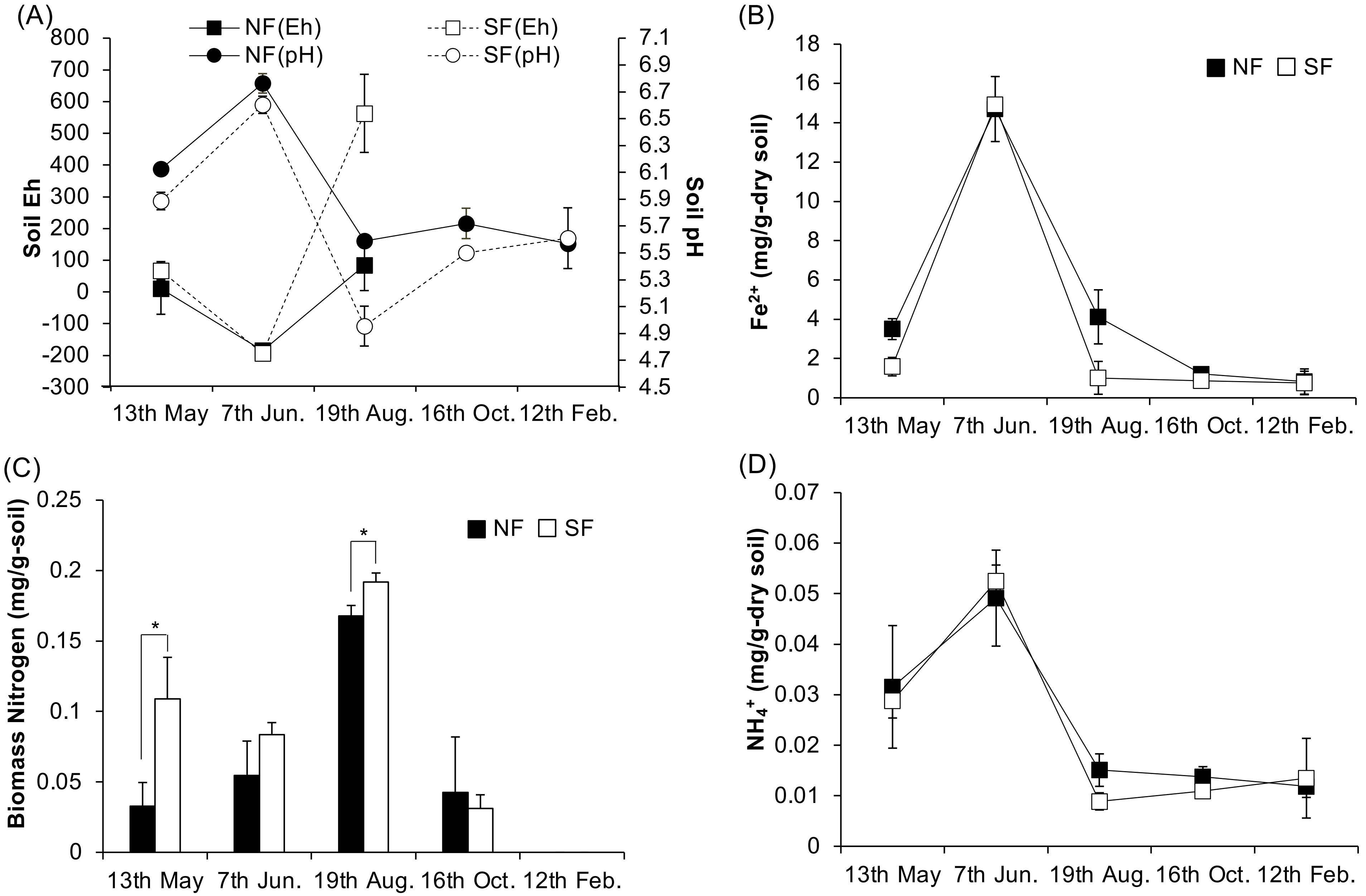


**Fig. S1 Transitions of the soil pH and the soil redox potential (Eh) (A), $Fe^{2+}$ concentration(B), biomass nitrogen (C), and $NH_4^+$ concentration (D) in both plot soils. NF, no fertilization; SF, standard fertilization.**

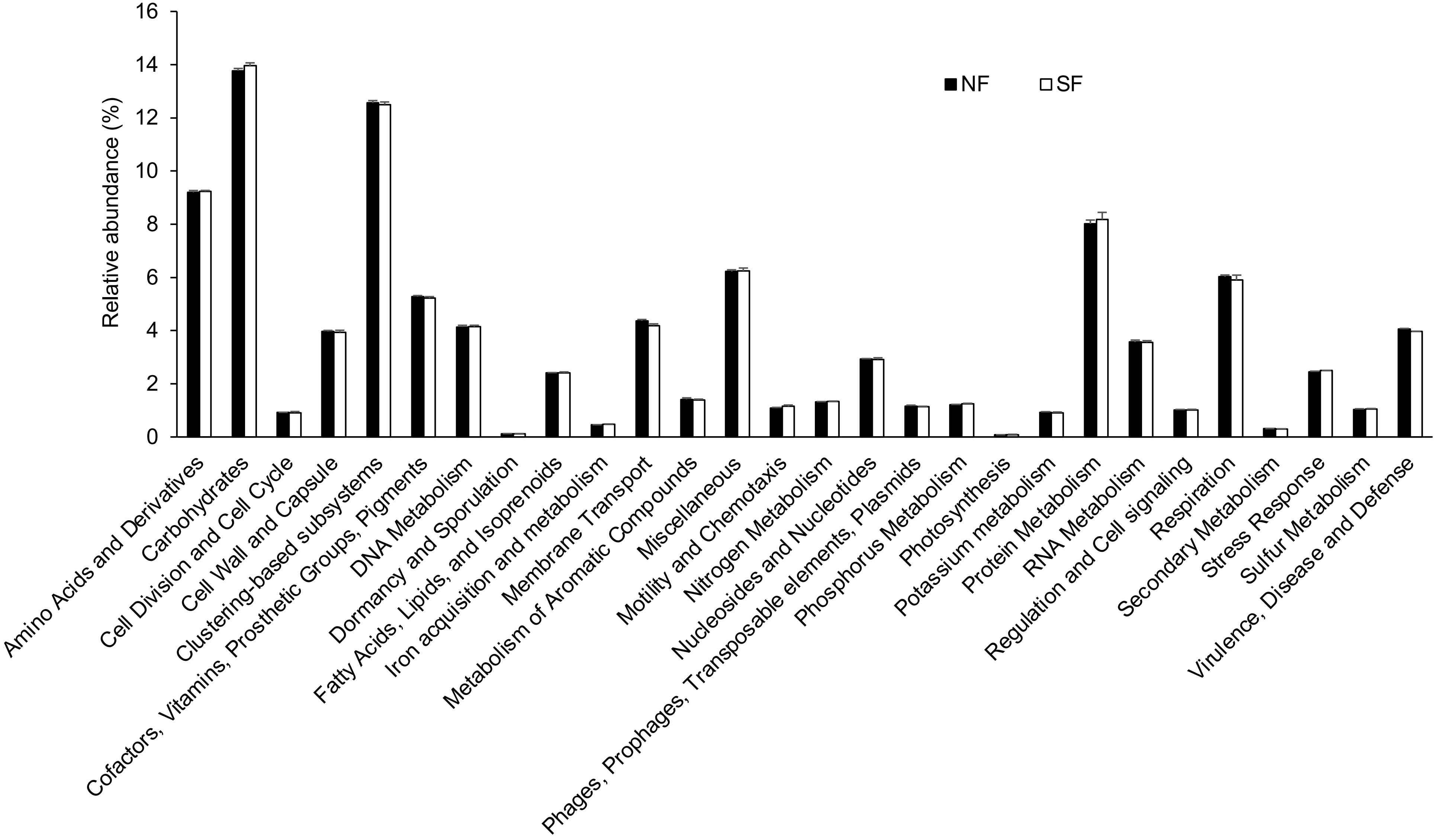


**Fig. S2 Composition of functional gene categories based on metagenomic analysis of both plot soils. NF, no fertilization; SF, standard fertilization**
