# Supplementary material for: Biological nitrogen fixation in the long-term nitrogen-fertilized and unfertilized paddy fields, with special reference to diazotrophic iron-reducing bacteria
Source: Arch Microbiol. 2023 Jul 20;205(8):291. doi: 10.1007/s00203-023-03631-8 (PMC10359436; doi:10.1007/s00203-023-03631-8)
Supplement: Supplementary file 2 — Supplementary file2 (DOCX 19 KB) [file 203_2023_3631_MOESM2_ESM.docx]

Table S1 Physicochemical characteristics of both plot soils

|  | Soil type | Free iron oxide  (%) | Total C  (g/kg-soil) | Total N  (g/kg-soil) | C/N |
| --- | --- | --- | --- | --- | --- |
| NF | Gray Lowland Soil | 1.4 | 11.0 | 1.1 | 9.7 |
| SF | Gray Lowland Soil | 1.5 | 11.0 | 1.09 | 10.1 |

NF, no fertilization; SF, standard fertilization

Table S2 Summary of metagenomics of soil samples collected from both plots on 7th June

|  | NF | | | SF | | |
| --- | --- | --- | --- | --- | --- | --- |
| Sample No. | 1 | 2 | 3 | 1 | 2 | 3 |
| MG-RAST ID | mgm4903913.3 | mgm4903914.3 | mgm4903915.3 | mgm4903904.3 | mgm4903905.3 | mgm4903912.3 |
| Sequence | 1764405 | 2186434 | 2225168 | 1864174 | 1163996 | 1487066 |
| Sequence length | 275 ± 96 bp | 275 ± 96 bp | 257 ± 106 bp | 273 ± 96 bp | 293 ± 50 bp | 270 ± 95 bp |
| Idetified protein | 731688 | 894629 | 861635 | 768486 | 456830 | 618348 |
| Identified rRNA | 1006 | 1350 | 1232 | 1176 | 640 | 943 |

NF, no fertilization; SF, standard fertilization
